# Microsatellite Mutation Rate during Allohexaploidization of Newly Resynthesized Wheat

**DOI:** 10.3390/ijms131012533

**Published:** 2012-10-01

**Authors:** Jiangtao Luo, Ming Hao, Li Zhang, Jixiang Chen, Lianquan Zhang, Zhongwei Yuan, Zehong Yan, Youliang Zheng, Huaigang Zhang, Yang Yen, Dengcai Liu

**Affiliations:** 1 Triticeae Research Institute, Sichuan Agricultural University, Wenjiang 611130, China; E-Mails: luojiangtao1987@126.com (J.L.); haoming.1987@yahoo.com.cn (M.H.); zhangli19830116@hotmail.com (L.Z.); chenjixiang1986@126.com (J.C.); zhanglianquan1977@yahoo.com (L.Z.); yzwyvon2004@126.com (Z.Y.); zhyan104@163.com (Z.Y.); ylzheng@sicau.edu.cn (Y.Z.); 2 Key Laboratory of Adaptation and Evolution of Plateau Biota, Northwest Institute of Plateau Biology, the Chinese Academy of Sciences, Xining 810001, China; E-Mail: hgzhang@nwipb.ac.cn; 3 Department of Biology and Microbiology, South Dakota State University, Brookings, SD 57007, USA; E-Mail: yang.yen@sdstate.edu

**Keywords:** allopolyploidy, distant hybridization, microsatellite evolution, wheat

## Abstract

Simple sequence repeats (SSRs, also known as microsatellites) are known to be mutational hotspots in genomes. DNA rearrangements have also been reported to accompany allopolyploidization. A study of the effect of allopolyploidization on SSR mutation is therefore important for understanding the origin and evolutionary dynamics of SSRs in allopolyploids. Three synthesized double haploid (SynDH) populations were made from 241 interspecific F_1_ haploid hybrids between *Triticum turgidum* L. and *Aegilops tauschii* (Coss.) through spontaneous chromosome doubling via unreduced gametes. Mutation events were studied at 160 SSR loci in the S_1_ generation (the first generation after chromosome doubling) of the three SynDH populations. Of the 148260 SSR alleles investigated in S_1_ generation, only one mutation (changed number of repeats) was confirmed with a mutation rate of 6.74 × 10^−6^. This mutation most likely occurred in the respective F_1_ hybrid. In comparison with previously reported data, our results suggested that allohexaploidization of wheat did not increase SSR mutation rate.

## 1. Introduction

Simple sequence repeats (SSRs, also known as microsatellites) are a class of DNA that is composed of short tandem repeats of a basic motif of one to six nucleotides. SSRs are ubiquitous in genomes. The nature of motif repetition makes SSRs inherently instable and prone to mutation by mechanisms of replication slippage or unequal crossing over [1]. Because of their high mutability, SSRs are thought to play a significant role in genome evolution [2]. Meanwhile, their abundance and high polymorphism make SSRs a popular molecular marker system in genetic studies and breeding programs as well [3].

Since it is a critical parameter in models of population genetics, the SSR mutation rate has been estimated in numerous studies [1,4]. The estimated average SSR mutation rate per allele and per generation varied greatly among species. For example, the rate was estimated as 10^−6^ in fruit fly [5], 10^−2^ in human [6] or about 10^−3^~10^−4^ in crops like durum wheat [7], common wheat [4], and maize [8]. Exposed to external stresses such as irradiation, oxidative damage, *in vitro* culture *etc.* will increase SSR mutation rates [2].

Allopolyploidization is an important evolutionary process that merges two distinct genomes into the nucleus to form an allopolyploid species. This process is particular important to higher plants. The merger of two distinct genomes presents a “genomic shock”, as McClintock [9] described, to which plants respond with a variety of genomic changes. DNA sequence rearrangements accompanied with allopolyploidization have been reported in various allopolyploid plant models, such as wheat [10–13], *Brassica* [14–18], *Triticale* [19–21] and *Arabidopsis* [22,23]. Allopolyploidization involves two steps: interspecific hybridization and chromosome doubling. It is interesting to know whether the two steps can accelerate SSR mutations. Such a study, however, is difficult since the sample size of independent allopolyploidization events needs to be large enough to reach a sufficient accuracy in detection of mutation events and conducting the two steps in experimental condition is very labor- and time-consuming. Utilizing genes that promote formation of unreduced gametes in haploids is a way to facilitate such a study [24].

**Figure 1:**
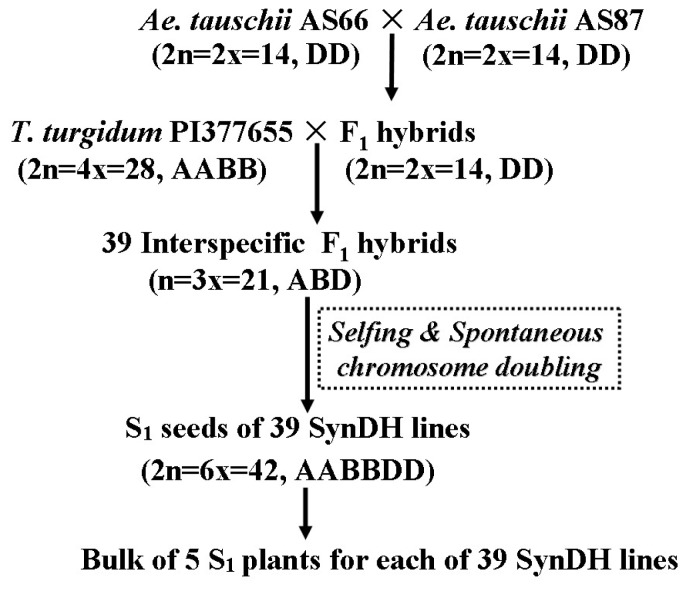
The production and pedigree of synthetic double haploid population SynDH3 used in this study. *Ae: Aegilops; T: Triticum*; SynDH: synthesized double haploid.

In the present study we investigated SSR mutation events that existed in the 241 S_1_ (the first generation after chromosome doubling) lines from three synthesized double haploid (SynDH) populations. Each of these SynDH lines was derived from an independently obtained interspecific F_1_ haploid hybrid between *Triticum turgidum* L. and *Aegilops tauschii* Coss (Table 1; Figure 1). These 241 SynDH hexaploid wheat lines represent 241 independent allopolyploidization events and therefore, are excellent materials for estimating SSR mutation rate during this important evolutionary event.

## 2. Results and Discussion

SSR mutations are predominantly manifested in changing numbers of the repeat motif. Hence, our investigation of SSR mutants and thus the estimation of SSR mutation rate were focused on variation of the repeat numbers at each SSR locus using bulked DNA samples from five randomly selected S_1_ plants per SynDH line. The five S_1_ plants from each SynDH line are expected to be genetically identical since they were derived from a single F_1_ hybrid (Figure 1).

**Figure 2:**
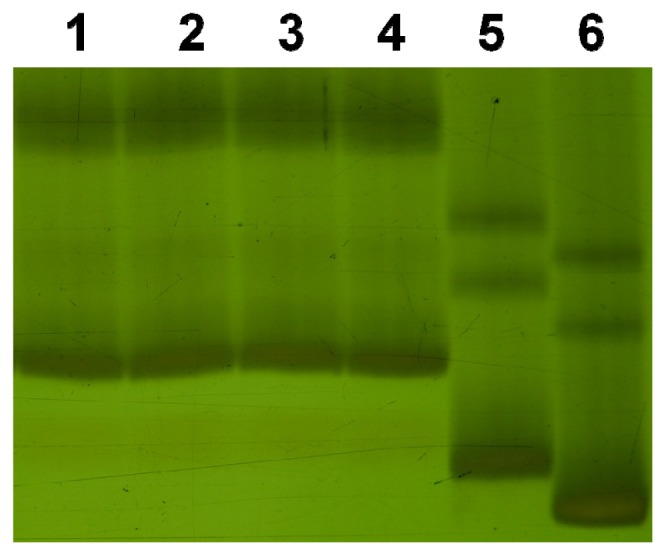
SSR amplification patterns of marker *Xcfd4*. The samples were two of SynDH1 lines (**1**, **2**); DNA mixture of their three parents (AS313, LDN and AS60) with a ratio of 1:1:1 (**3**); *Aegilops tauschii* line AS60 (**4**); *Triticum turgidum* parents LDN (**5**) and AS313 (**6**).

**Figure 3:**
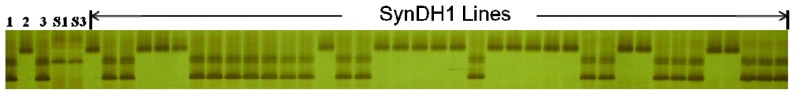
SSR amplification patterns of marker *Xwmc312*. Compared to their parents AS60 (**1**); LDN (**2**) and AS313 (**3**); **S_1_** (lane S1) and **S_3_** (lane S3) of SynDH1 line 61 showed novel SSR patterns. AS313 and AS60 had same SSR pattern. SSRs from AS60 (1) were absent in SynDH1 line 61 (S1 and S3) and these SynDH1 lines carrying LDN allele. The SSRs in other SynDH1 lines should be the products of AS313 allele (3) rather than AS60 (1) due to a competition for PCR amplification.

A total of 160 SSR markers that were polymorphic between the parents were used in this study (Table 1). Of them, 148 were mapped to particular loci of the SynDH lines (Table S1). The chromosome locations of the remaining 12 were determined based on the information from GrainGenes database. Of the three SynDH populations, a total of 148260 SSR alleles were recognized and investigated. As expected, the amplification pattern in a SynDH S_1_ line was a combination of its parents’ for 158 markers (Figure S1 showing an example image for marker *Xbarc137*). However, the expected SSR patterns from *T. turgidum* parents for marker *Xcfd4* (Figure 2) and from *Ae. tauschii* for marker *Xwmc312* (Figure 3) were lost in the analyzed SynDH1 lines. However, the losses were also observed in the amplification patterns of DNA mixtures of its parents. Apparently, the observed losses did not result from SSR mutations but most likely from a competition for PCR amplification between the parental homoeo-alleles.

**Figure 4:**
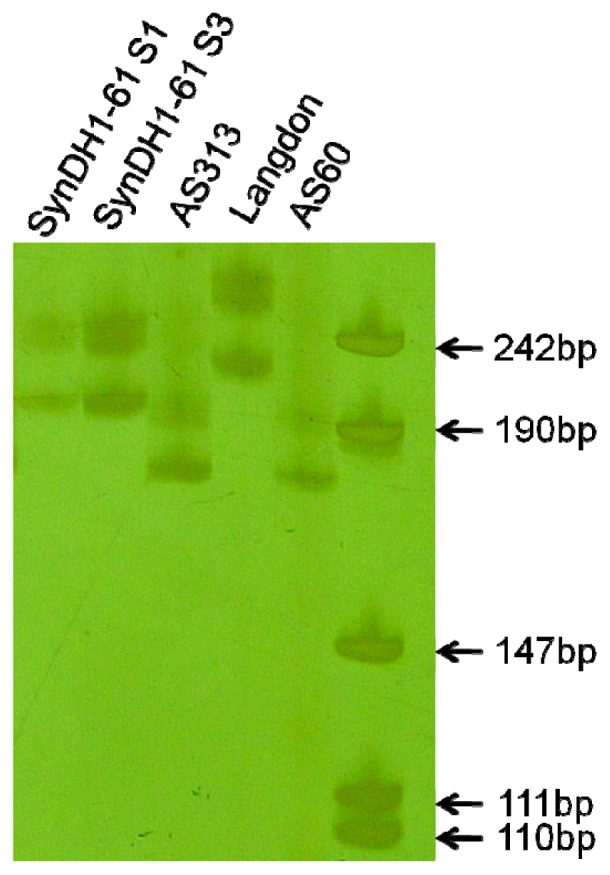
SSR amplification patterns of marker *Xwmc312*. Compared with their parents AS60, Langdon and AS313, S1 and S3 of SynDH1 line 61 showed novel SSR patterns. The 6% polyacrylamide gel used in this study that can detect 1 bp difference between 110 and 111 bp products.

SSR gain was only observed in the line 61 of SynDH1 population at locus *Xwmc312*. To make sure that this gain mutation is true, we repeated the PCR assay eight times and then assayed the respective S_3_ plants eight times. We were able to confirm the observation in each of these assays (Figures 3,4), suggesting that this SSR mutation was homozygous in the S_1_ and has been stably transmitted into the offspring. This SSR mutation most likely took place in the respective haploid F_1_ and then homologized in the S_1_ via the union of unreduced gametes. SSR marker *Xwmc312* on 1A was mapped with the SynDH1 population and was found to be flanked with SSR markers *Xbarc148*, *Xgwm357* and *Xwmc83*, high-molecular-weight glutenin subunit *Glu-A1*, and Diversity Arrays Technology (DArT) loci *wPt-4658*, *wPt-5316* and *wPt-8016* (data not shown). Since all these flanking loci in SynDH1 line 61 were derived from LDN, this novel SSR was apparently mutated from the LDN allele.

**Figure 5:**
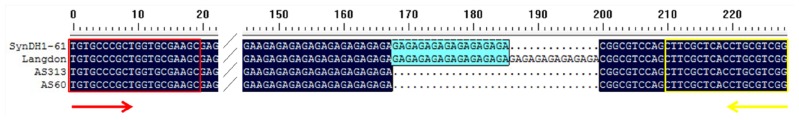
Sequence alignment of the DNA sequence of the *Xwmc312* alleles cloned from SynDH1-61 (Genbank number JQ404447), LDN (JQ404446), AS313 (JQ404445) and AS60 (JQ404444). The red rectangle with an arrow indicated forward primer region and the yellow rectangle with an arrow indicated reverse primer region. The diagonal represented the ellipsis sequences.

This novel SSR was confirmed by analyzing the sequences of the *Xwmc312* alleles cloned from SynDH1 line 61 and its parents. A total of 50 clones were sequenced and analyzed. All of the clones contained the same SSR flanking sequences of 173 bp and differed only in the number of the GA repeats they had. However, we failed to get a clean sequence for each line, which may be caused by PCR-generated stutter [4,7]. Based on the frequency of appearance of the SSR sequences for each line (Table 2), the approximate lengths of the *Xwmc312* alleles of AS313, AS60, LDN, and SynDH line 61 were estimated as 197 bp (12 repeats, Genbank number JQ404445), 197 bp (12 repeats, JQ404444), 229 bp (28 repeats, JQ404446), and 215 bp (21 repeats, JQ404447), respectively (Figure 5). It seems that the novel allele in SynDH1 line 61 was originated by deleting seven GA repeats from the LDN allele.

The SSR mutation rate S_1_ generation was estimated by dividing the number of the observed mutant allele (*o*) by the total number of possible mutant alleles (*n*). The *n* number for S_1_ generation of each SynDH population was estimated based on the total observed SSR alleles at all of the loci (Table 1). The estimated average mutation rates were, therefore, 1/93790 = 1.06 × 10^−5^ for SynDH1 S_1_ and 1/148260 = 6.74 × 10^−6^ for the three S_1_ populations. The estimated SSR mutation rates were lower than the *ca.* 10^−3^~10^−4^ per allele per generation that were previously estimated in durum wheat [7] and common wheat [4]. Except for marker *Xcfd161*, repeat motif of markers used in this study has a length of at least 2 bp (Table S1). We used 6% polyacrylamide gels that can detect 1 bp difference between 110 and 111 bp products (Figure 4). Given the PCR-stutter and the pooled screening with a bulk DNA of five plants for each line, however, we cannot exclude that the low mutation rate resulted from underestimated small variations of 1 or 2 bp. Anyway, this study did not provide evidence that allopolyploidization during resynthesizing hexaploid wheat increased SSR mutation rate, which was agreed with that SSR sequences are highly conserved during allopolyploidization of hexaploid wheat [25] and of *Brassica* [15].

As mentioned earlier in this communication, allopolyploidization-accompanied DNA sequence rearrangements have been reported in some allopolyploids. However, no evidence for sequence variation was found in re-synthesized cotton [26] and some allohexaploid wheats [25,27,28]. These different conclusions may be a result of different genome combinations and/or different DNA types revealed by different technical methods. The effects of genome combination on inducing variations have shown by that hybrids of wheat with rye have higher rate of DNA sequence changes than hybrids of wheat with other related species [19,20]. This might be due to the fact that rye is more genetically distant to wheat than other species used. Effects by DNA types have been also demonstrated by Lukens *et al.* [15] with the allopolyploid *Brassica*. Their results indicate no variation for SSR, low frequent variations for restriction fragment length polymorphism (RFLP), but high frequent variations for DNA methylation. The methods used to assess DNA variation based on methyl-sensitive or nonsensitive restriction enzymes also revealed a lot of variations in allopolyploid wheat [11–13]. However, SSR sequences are highly conserved in allohexaploid wheat [25].

The different conclusions from different DNA types may be finally attributed to different mutation mechanisms. Ellegren [1] proposed two mechanisms for SSR mutation: DNA polymerase slippage and unequal recombination. The former usually leads to a single novel allele and the latter should result in co-existence of alleles of different lengths. It seemed that the mutant *Xwmc312* allele observed in this study might result from DNA polymerase slippage, although the unequal recombination model could not be completely excluded [29]. The highly conserved SSRs during allopolyploidization of wheat may indicate that enzymes for replication slippage are conserved among A, B, and D genomes of its parent *T. turgidum* and *Ae. tauschii*. Comparing these enzymes among different species and their relationships with SSR mutation rates are necessary to further test this hypothesis.

## 3. Experimental Section

### 3.1. Plant Materials

Plant materials used in this study included *Triticum turgidum* ssp. *durum* line Langdon (abbreviated as LDN), ssp. *dicoccon* line PI377655, ssp. *turgidum* lines AS313 and AS2255, *Aegilops tauschii* accessions AS60, AS66 and AS87, and their synthesizing double haploid populations SynDH1, SynDH2, and SynDH3 (Table 1). These SynDHs were obtained by spontaneous chromosome doubling with the help of unreduced gametes [24,30]. Tetraploid F_1_ hybrids of LDN × AS313 and LDN × AS2255 were pollinated by *Ae. tauschii* AS60 to form triploid F_1_ hybrids with genomes ABD, respectively. The S_1_ seeds for SynDH1 and SynDH2 populations were obtained by selfing the LDN/AS313//AS60 and LDN/AS2255//AS60 triploid F_1_ hybrid plants, respectively [24]. Similarly, S_1_ seeds for SynDH3 population were obtained from the triploid F_1_s between AS66 × AS87 and PI377655 (Figure 1). All the triploid F_1_ hybrids for the three populations spontaneously produced S_1_ doubled haploid seeds since all the three *T. turgidum* parents have genes for the formation of unreduced gametes in haploid status [24,30]. S_1_ seeds from a single triploid F_1_ plant formed a SynDH line. Only A- and B-genome chromosomes between *T. turgidum* lines were involved in genetic recombination for SynDH1 and SynDH2 populations [24], while only D-genome chromosomes between *Ae. tauschii* lines were involved in recombination for SynDH3 (Figure 1). No embryo rescue technique or hormone treatment was applied for the production of triploid F_1_ hybrids. Five S_1_ plants were grown for each SynDH line in order to obtain a bulked leaf sample for genomic DNA isolation.

### 3.2. Simple Sequence Repeats (SSRs) Analysis

Leaf samples were collected at heading stage from parents and the SynDH lines, ground in liquid nitrogen and used for DNA isolation with a modified 2 × CTAB method [31]. For each SynDH line, total genomic DNA was isolated from a leaf sample bulked from five S_1_ plants [24], which represented independent chromosome doubling events that are expected to be genetically identical.

Eight-one polymorphic SSR markers between the two *T. turgidum* parents of LDN and AS313 were used in investigation of SynDH1 population (Table S1). Of them, 12 on chromosome 3B or 6B that were polymorphic between the parents LDN and AS2255 were also used in the study of SynDH2 population (Table S1). These SSR markers have been mapped on A- or B-genome chromosome, respectively. Seventy-nine markers from D genome chromosomes were used in analyzing SynDH3 population (Table S1). They were polymorphic between *Ae. tauschii* parents AS66 and AS87 and, respectively, represent each D-genome chromosome [32–37]. PCR reactions were performed as previously described [24]. All of the SynDH samples were run together with their parent DNA as the controls. The amplified fragments were separated by electrophoresis in 6% polyacrylamide denaturing gels (8 M urea, 35-cm-wide × 29-cm-long) and visualized with silver-staining method [31]. Run the gels in 1× TBE buffer at 75 W and 1600 V constant power until the indicator bromophenol blue reached the bottom.

For the survey of SSR mutations, amplification patterns observed in the SynDH lines were compared with those of their respective parents. The presence of a SSR fragment in a parent that was absent from the corresponding SynDH line was recognized as a loss. The presence of a SSR fragment in a SynDH line that was not observed in the parents was recognized as a gain. Accordingly, a marker was considered as non-changed if its patterns in a SynDH line were a combination of the parents’. By contrast, a marker was considered as changed if an unexpected amplification fragment appeared in a SynDH line. All PCR reactions that generated unexpected changes of either a loss- or a gain-type or an unclear fragment were repeated for confirmation.

### 3.3. Cloning and Sequencing

Mutant alleles and their parent alleles were re-amplified, cloned, and sequenced to reconfirm the mutation and to determine its nature. The re-amplified PCR products were separated by 6% polyacrylamide denaturing gels. The target fragments were cut and boiled in 30 μL ddH_2_O for 10 min and then centrifuged at 4000× *g* for 10 min. The upper liquid was used to conduct the second PCR reaction and the PCR products were then separated on 2.5% agarose gels followed by purification using Gel DNA Recovery Kit (PUEX, USA.). The purified PCR products were cloned into pMD19-T vector using cloning kit from TaKaRa Biotechnology (Dalian, China) Co., Ltd. [38]. Positive clones were identified and then sequenced by BGI (Beijing, China). Sequence alignments were conducted using the DNAMAN 6.0 Demo software (Lynnon Biosoft).

### 3.4. Estimation of SSR Mutation Rate

In this study, a SSR amplification pattern from a parent was estimated as an SSR allele. This estimation was supported by our mapping results that most of the 160 markers used in this study were mapped as a single locus on a chromosome based on their genotyping using the three SynDH populations (Table S1). Based on the observed amplification pattern in SynDHs, the scored SSR markers were further classified as two types: (A) amplifying SSR products at two SSR alleles in a SynDH line, one from *T. turgidum* and the other from *Ae. tauschii*; (B) only amplifying SSR products at one of the two SSR alleles from *T. turgidum* and *Ae. tauschii*.

The mutation rate was calculated by dividing the allele number of observed mutants by the allele number (*n*) of total possible mutation events, which were calculated by summarizing all the alleles from all the plants in a SynDH population. Depending upon the nature of a SSR marker, an S_1_ plant with AABBDD genome composition could reveal two homologous alleles from *T. turgidum* and two from *Ae. tauschii* or only reveal two homologous alleles from one of the parents. Meanwhile, a bulked DNA of five S_1_ plants for each SynDH line was used. Therefore, for a S_1_ population, the *n* was estimated by the following equation:

n=(4 A+2 B)×N×5

where:

*A*: number of SSR markers that revealed four alleles

*B*: number of SSR markers that revealed two alleles

*N*: number of S_1_ lines.

## 4. Conclusions

In this study, mutation events were studied at 160 SSR loci in S_1_ generation of the three SynDH populations. Of the 148260 SSR alleles investigated in S_1_ generation, only one mutation (changed number of repeats) was confirmed with a mutation rate of 6.74 × 10^−6^. This SSR mutation was homozygous in the analyzed S_1_ plants of line SynDH1-61. Therefore, this mutation most likely occurred in the respective F_1_ hybrid and then homologized in the S_1_ via the union of unreduced gametes. Compared with previously reported data as regards durum wheat or common wheat itself, our results suggested that allohexaploidization of wheat did not increase the SSR mutation rate.

## Supplementary Materials



## Figures and Tables

**Table 1:** Numbers of simple sequence repeats (SSR) markers scored and their estimated alleles in three populations.

Double haploid populations (hybrid combinations)	Hybrids of haploid (triploid) F_1_ hybrids		No. S_1_ lines analyzed *	No. of scored markers **	No. of estimated S_1_ alleles
	Female	Male			
SynDH1 (LDN/AS313//AS60)	F_1_ hybrids between *T. turgidum* LDN and AS313	*Ae. tauschii* AS60	113	81	93790
SynDH2 (LDN/AS2255//AS60)	F_1_ hybrids between *T. turgidum* LDN and AS2255	*Ae. tauschii* AS60	89	12	11570
SynDH3 (PI377655//AS66/AS87)	*T. turgidum* PI377655	hybrids between *Ae. tauschii* AS66 and AS87	39	79	42900
Total	-	-	241	160	148260

* Each of these S_1_ SynDH lines was derived from an independently obtained interspecific F_1_ haploid hybrid between *Triticum turgidum* L. and *Aegilops tauschii* Coss. A bulked DNA sample from five randomly selected S_1_ plants per SynDH line was used in investigation of SSR mutation events. The five S_1_ plants from each SynDH line are expected to be genetically identical since they were derived from a single F_1_ hybrid;

** Of the 81 markers used in SynDH1, 12 were also used in SynDH2. Therefore, a total of 160 SSR markers were used.

**Table 2:** The distribution of SSR sequence length for marker *Xwmc312.*

Materials	No. of sequenced clones	Number of clones with different sequence length												

		193 (bp)	195 (bp)	197 (bp)	199 (bp)	213 (bp)	215 (bp)	217 (bp)	219 (bp)	223 (bp)	227 (bp)	229 (bp)	231 (bp)	233 (bp)
AS313	8	-	1	7	-	-	-	-	-	-	-	-	-	-
Langdon	9	-	-	-	-	-	-	-	-	1	2	4	1	1
AS60	9	1	1	6	1	-	-	-	-	-	-	-	-	-
SynDH1-61 (S1)	13	-	-	-	-	-	9	3	1	-	-	-	-	-
SynDH1-61 (S2)	11	-	-	1	-	3	4	3	-	-	-	-	-	-
