# Supplementary material for: Microsatellite Mutation Rate during Allohexaploidization of Newly Resynthesized Wheat
Source: Int J Mol Sci. 2012 Oct 1;13(10):12533–43. doi: 10.3390/ijms131012533 (PMC3497285; doi:10.3390/ijms131012533)
Supplement: Supplementary file 1 [file ijms-13-12533-s001.pdf]

Supplementary Information

Table S1. Markers used in this study.

Markers scored in SynDH1 and SynDH2			Markers scored in SynDH3		
**Marker**	**Chromosome**	**Repeat Motif**	**Marker**	**Chromosome**	**Repeat Motif**
Xwmc312	1A	(GA)	*Xwmc36*	1D	(CA)(GT)
Xbarc17	1A	(TAA)	*Xgwm642*	1D	(GT)
Xbarc148	1A	(CT)	*Xwmc609*	1D	(GA)
Xbarc287	1A	(TTA)	*Xcfd15*	1D	(CT)(TGTA)
Xgwm357	1A	(GA)	*Xgwm337*	1D	(CT)(CACT)(CA)
Xwmc83	1A	(GT)	*Xgpw7109*	1D	(GT)
Xbarc187	1B	(CT)	*Xgpw357*	1D	(CA)
Xbarc137	1B	(CT)	*Xgpw7082*	1D	(TGT)
Xbarc81	1B	(ATG)	*Xgdm126*	1D	(CA)
Xwmc406	1B	(CA)	*Xcfd282*	1D	(TG)
Xbarc302	1B	(ATT)	*Xcfd61*	1D	(CACAA)(AC)
Xgwm11	1B	(TA)CATA(CA)(TA)	*Xcfd27*	1D	(CA)
Xgwm273	1B	(GA)	*Xbarc99*	1D	(TAG)(TAA)
Xbarc61	1B	(TAGA)	*Xcfd83*	1D	(TG)
Xwmc128	1B	(GT)	*Xcfd161*	2D	(G)(A)
Xgwm153	1B	(GA)	*Xgwm157*	2D	(CT)
Xwmc420	2A	(GT)	*Xgwm484*	2D	(CT)
Xwmc522	2A	(CT)(TC)	*Xgdm77*	2D	(GA)
Xgwm526	2A	(CT)	*Xgwm320*	2D	(GT)(GA)
Xgwm265	2A	(GT)	*Xgdm93*	2D	(GA)
Xwmc658	2A	(GA)	*Xgwm261*	2D	(CT)
Xwmc317	2B	(GT)	*Xgwm539*	2D	(GA)
Xgwm120	2B	(CT)(CA)	*Xwmc41*	2D	(GA) (GCC)
Xwmc592	2B	(GA)	*Xgwm382*	2D	(GA)
Xbarc19	3A	(TAA)	*Xcfd44*	2D	(CT)
Xgwm376	3B	(CA)(GA)	*Xcfd233*	2D	(GA)
Xgwm493	3B	CA)	*Xcfd53*	2D	(CT)(CA)
Xgwm389	3B	(CT)(GT)	*Xgwm71*	3D	(GT)
Xgwm284	3B	(GA)	*Xcfd9*	3D	(TC)
Xgwm181	3B	(GA)	*Xgwm161*	3D	(CT)
Xgwm285	3B	(GA)	*Xcfd152*	3D	(TC)
Xgpw7774	3B	(AGC)	*Xgwm314*	3D	(CT)
Xbarc139	3B	(CA)	*Xgwm383*	3D	(GT)
Xgpw1146	3B	(CT)	*Xcfd35*	3D	(GT)(CT)
Xgpw7148	3B	(AG)	*Xgwm664*	3D	(GA)
Xgwm299	3B	(GA)(TAG)	*Xgdm72*	3D	(CT)
Xgwm247	3B	(GA)	*Xbarc323*	3D	(CT)
Xgpw3134	3B	(GA)	*Xwmc457*	4D	(CA)
Xgpw3248	3B	(AG)	*Xwmc331*	4D	(CA) (CT)
Xwmc675	3B	(GA)	*Xwmc720*	4D	(GA)
Xgwm340	3B	(GA)	*Xwmc473*	4D	(CA)
Xwmc313	4A	(CA)	*Xgdm61*	4D	(GT)

Table S1. *Cont.*

**Markers scored in SynDH1 and SynDH2**			**Markers scored in SynDH3**		
Xwmc468	4A	(CT)	*Xwmc285*	4D	(CA) (CA)
Xgwm610	4A	(GA)	*Xgdm34*	4D	(GA)
Xgwm251	4B	(CA)	*Xgwm192*	4D	(CT)
Xgwm595	5A	(GA)	*Xwmc443*	5D	(CT)
Xbarc151	5A/7A	(CT)	*Xgwm212*	5D	(CT)
Xbarc142	5B	(CT)	*Xgdm63*	5D	(CT)
Xgwm499	5B	(GA)	*Xgwm190*	5D	(CT)
Xgwm544	5B	(CT)(ATCT)(CT)	*Xcfd12*	5D	(CTT)
Xwmc28	5B	GT)	*Xcfd3*	5D	(CA)
Xgwm67	5B	(CA)	*Xwmc318*	5D	(GT)
Xgwm604	5B	(GA)	*Xgwm639*	5D	(GA)
Xbarc232	5B	(CT)	*Xbarc347*	5D	(CT)
Xgwm234	5B	(CT)(CA)	*Xcfd57*	5D	(GA)
Xbarc59	5B	(TAGA)	*Xwmc233*	5D	(CA)
Xbarc4	5B	(TTA)	*Xwmc357*	5D	(GA) (GT)
Xgwm356	6A	(GA)	*Xgwm325*	6D	(CT)
Xbarc3	6A	(CCT)	*Xgwm469*	6D	(CT)(CA)
Xgwm570	6A	(CT)(GT)	*Xwmc416*	6D	(CA)(CA)(CA)
Xgwm550	6A	(CT)(GT)	*Xgdm36*	6D	(GT)
Xwmc553	6A	(CA)	*Xcfd42*	6D	(GA)
Xgwm133	6B	(CT)	*Xcfd49*	6D	(GA)
Xwmc398	6B	(GT)	*Xbarc173*	6D	(ATT)(TAG)(CAA)
Xbarc178	6B	(ATT)	*Xbarc96*	6D	(TAA)
Xwmc494	6B	(GT)	*Xcfd33*	6D	(CA)
Xwmc537	6B	(CA) (CT)	*Xgdm99*	7D	(GA)
Xbarc79	6B	(TAGA)(TC)	*Xgwm111*	7D	(CT)(GT)
Xwmc487	6B	(CA)	*Xgwm428*	7D	(GA)
Xgwm626	6B	(CT)(GT)	*Xwmc94*	7D	(GT)
Xgpw1079	6B	(GT)	*Xwmc438*	7D	(CA)(CA)
Xgpw3153	6B	(AC)	*Xgdm86*	7D	(CT)
Xbarc127	7A	(CT)	*Xwmc824*	7D	(GA)
Xgwm130	7A	(GT)	*Xcfd14*	7D	(TC)
Xgwm276	7A	(CT)	*Xwmc506*	7D	(GA) (GA)
Xwmc346	7A	(GA) (GT)	*Xcfd31*	7D	(GA)
Xcfa2257	7A	(TG)	*Xpsp3123*	7D	(TC)
Xbarc72	7B	(CT)(TCTA)(TC)	*Xbarc172*	7D	(ATT)
Xwmc76	7B	(GT)	*Xcfd68*	7D	(GT)
Xwmc364	7B	(CA)	-	-	-
Xgwm333	7B	(GA)	-	-	-

Of the 160 markers, 148 were mapped using the SynDH populations used in this study. The mapped locations of the remaining 12 markers (highlighted as red) were determined on the basis of information from GrainGenes (http://wheat.pw.usda.gov/cgi-bin/graingenes/browse.cgi?class=marker). The 12 markers highlighted as yellow were used in both SynDH1 and SynDH2 populations. The repeat motifs highlighted as blue were obtained in this study by SSR sequencing.

Figure S1. SSR amplification patterns of marker *Xbarc137* in some SynDH1 lines.

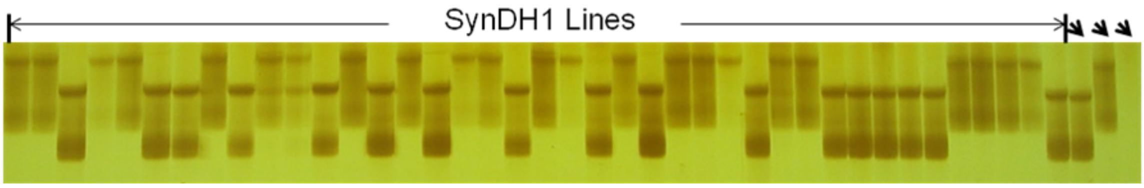

SSR amplification patterns of marker Xbarc137 in some SynDH1 lines. The arrows show the three parents AS313, Langdon and AS60 from left to right, respectively.

© 2012 by the authors; licensee MDPI, Basel, Switzerland. This article is an open access article distributed under the terms and conditions of the Creative Commons Attribution license (http://creativecommons.org/licenses/by/3.0/).
